# Assessment of potential nephrotoxicity biomarkers after [^177^Lu]Lu-DOTA-(Tyr^3^)-octreotate administration and effects of antioxidant α_1_-microglobulin

**DOI:** 10.1038/s41598-026-61293-0

**Published:** 2026-07-16

**Authors:** Charlotte Ytterbrink, Daniella Pettersson, Emman Shubbar, Khalil Helou, Martin E. Johansson, Eva Forssell-Aronsson

**Affiliations:** 1 Department of Medical Radiation Sciences, Institute of Clinical Sciences, Sahlgrenska Academy, University of Gothenburg, Gothenburg, Sweden; 2 Sahlgrenska Center for Cancer Research, Sahlgrenska Academy, University of Gothenburg, Gothenburg, Sweden; 3 Department of Oncology, Institute of Clinical Sciences, Sahlgrenska Academy, University of Gothenburg, Gothenburg, Sweden; 4 Department of Laboratory Medicine, Institute of Biomedicine, Sahlgrenska Academy, University of Gothenburg, Gothenburg, Sweden; 5 Department of Medical Physics and Biomedical Engineering, Sahlgrenska University Hospital, Gothenburg, Sweden; 6 Department of Medical Radiation Sciences, University of Gothenburg, Sahlgrenska University Hospital, Gothenburg, SE-413 45 Sweden

**Keywords:** Alpha-1-microglobulin, RBP4, Cystatin C, KIM-1, Kidney toxicity, Radionuclide therapy, Biomarkers, Nephrology

## Abstract

Patients with neuroendocrine tumours may receive higher total amount of [^177^Lu]Lu-DOTA-(Tyr^3^)-octreotate and achieve better tumor control if the risk of kidney toxicity could be predicted individually for each patient. One strategy for such a prediction includes the evaluation of early responding biomarkers for kidney damage during treatment to decide the number of treatment cycles. The aims of this study were to (1) evaluate if urinary RBP4, NGAL, creatinine and cystatin C levels may serve as early responding biomarkers for kidney damage, (2) assess the expression of NGAL, KIM-1, CDKN1A, S100A6 and ADIPOQ in kidney tissue to validate their use as histological indicators of toxicity, and to (3) examine the long-term effects of the proposed radioprotector α_1_-microglobulin (A1M) in mice exposed to [^177^Lu]Lu-DOTA-(Tyr3)-octreotate. Mice were injected with 90 or 150 MBq [^177^Lu]Lu-DOTA-(Tyr3)-octreotate with or without A1M. Urine was collected at several time-points, and the animals were sacrificed after 9 months. Urinary RBP4 increased from day 35 after injection of 150 MBq [^177^Lu]Lu-DOTA-(Tyr^3^)-octreotate, creatinine decreased from day 131 and cystatin C increased from day 223. KIM-1, CDKN1A and S100A6 showed a dose dependent kidney expression. Morphological signs of kidney injury were observed in mice injected with 150 MBq [^177^Lu]Lu-DOTA-(Tyr^3^)-octreotate. No clear kidney protective effect was detected when A1M was co-administered with [^177^Lu]Lu-DOTA-(Tyr^3^)-octreotate.

## Introduction

In molecular radiation therapy, the tumor cells are the target of irradiation, but irradiation of healthy tissues is inevitable after systemically administered radiopharmaceuticals. Risk of side-effects is the major dose-limiting factor. In patients treated with somatostatin receptor (SSTR) expressing neuroendocrine tumors (NETs) with [^177^Lu]Lu-DOTA-(Tyr^3^)-octreotate (Lutathera^®^), kidneys are one of the major risk organs^1^. ^177^Lu is mainly excreted via the urine, but due to tubular reabsorption some ^177^Lu will be retained in the kidneys^2^. Re-uptake is mediated by the megalin-cubilin complex and to some extent also by SSTRs expressed by tubular cells. Consequently, the undesired renal accumulation of ^177^Lu increases the absorbed dose to kidneys resulting in a higher risk of kidney toxicity, which occurs late, months or rather years, after [^177^Lu]Lu-DOTA-(Tyr^3^)-octreotate therapy.

The current clinical [^177^Lu]Lu-DOTA-(Tyr^3^)-octreotate treatment protocol includes 4 × 7.4 GBq cycles combined with slow infusion of the amino acids *L*-lysine and *L*-arginine during, under and after administration of the radiopharmaceutical for renal protection^1^. The purpose of the administration of these positively charged amino acids is to reduce the uptake and reabsorption in kidneys. Despite the success of [^177^Lu]Lu-DOTA-(Tyr^3^)-octreotate therapy with prolonged overall survival, cure rate is low and relapses are common. The side-effects are very few after 4 cycles and most patients would tolerate more cycles than 4 or given higher amounts of [^177^Lu]Lu-DOTA-(Tyr^3^)-octreotate without increasing the risk of side effects. Hence, most patients are undertreated and increasing the total amount of administrated [^177^Lu]Lu-DOTA-(Tyr^3^)-octreotate could increase the probability of better tumor control and higher cure rate^3,4^. The absorbed dose to kidneys per injected activity of [^177^Lu]Lu-DOTA-(Tyr^3^)-octreotate varies considerably between patients (0.33–2.4 Gy/GBq), implying that individualized treatment must be applied for best outcome for each patient^5^. Focusing on nephrotoxicity, there are several options for optimization of treatment: (1) to reduce the uptake and retention of ^177^Lu in the kidneys by better blocking agents than applied routinely today (described above); (2) to reduce the radiobiological effects from ^177^Lu exposure by combining [^177^Lu]Lu-DOTA-(Tyr^3^)-octreotate administration with a radioprotector; and (3) to adjust the number of treatment cycles to highest possible without increasing the risk of late nephrotoxicity. These methods can be used one by one or preferably combined if optimal treatment effects will be obtained.

For option two, α_1_-microglobulin (A1M) has been proposed^6,7^. A1M is an endogenous antioxidant that has been referred to as a “radical sink” and “tissue housekeeping protein”^8,9^. The potential radio-protective mechanisms of A1M are not fully understood but it has been suggested that the radical scavenging abilities of A1M can protect cells from harmful free radicals, induced by ionizing radiation^8–10^. The potential radio-protective abilities that have been presented, together with the similarity in distribution within the kidneys of A1M and ^177^Lu makes A1M an interesting candidate for kidney protection during [^177^Lu]Lu-DOTA-(Tyr^3^)-octreotate treatment^11^. Initial preclinical studies on the renal effects after co-administration of A1M with [^177^Lu]Lu-DOTA-(Tyr^3^)-octreotate resulted in a reduction of DNA double-strand breaks in the kidneys and longer overall survival. In similar studies of A1M and [^177^Lu]Lu-PSMA-617 the combination treatment showed better kidney function (estimated by [^99m^Tc]Tc-MAG3 scintigraphy) than with [^177^Lu]Lu-PSMA-617 alone at 3 months, but with no observed differences between the groups at 6 months^12–14^. Since the kidney is a late responding organ more long-term studies on the potential radio-protective effects of A1M on the kidney function are needed. Furthermore, conclusions from previous findings need to be validated using other analyzing methods for detection of kidney damage.

The use of sensitive and early-responding biomarkers of kidney function for indication of late nephrotoxicity is most valuable for option three with optimization of number of cycles. Early detection of renal impairment is not only a prerequisite for determining optimum number of cycles, it can also be used to identify cases when additional radio-protective measures, like administration of radioprotectors, should be taken. Today the gold standard method for monitoring kidney function is measurement of glomerular filtration rate (GFR). Blood or urinary creatinine and cystatin C levels are commonly used to reflect GFR^15,16^. Radiation-induced reduction in GFR often occurs late after [^177^Lu]Lu-DOTA-(Tyr^3^)-octreotate treatment, too late to guide the optimal number of cycles. To maximize the amount of [^177^Lu]Lu-DOTA-(Tyr^3^)-octreotate that can be administered, while keeping the risk for late nephrotoxicity low, reliable early-responding biomarkers, easily available in blood or urine, can indicate when to interrupt the treatment to avoid late nephrotoxicity. Since the retention of [^177^Lu]Lu-DOTA-(Tyr^3^)-octreotate seems to take place mainly in the proximal tubules, a biomarker related to the function of these tubules could be beneficial.

Furthermore, much is still not known about radiation induced kidney toxicity, and improved knowledge about the biological response is most valuable to find better options for radioprotection. Expression of biomarkers in the kidney tissue can give a better understanding of the biological processes induced and how to prevent development of tissue damage.

Neutrophil gelatinase-associated lipocalin (NGAL), kidney injury molecule 1 (KIM-1) and retinol binding protein 4 (RBP4) are among the most promising suggested biomarkers for early detection of kidney damage^15,17^. However, their usefulness for the prediction of radiation-induced kidney damage is not established. In our previous studies on potential biomarkers for irradiation induced kidney damage^18–22^, these proteins were suggested. In the transcriptomic studies, the transcripts of NGAL and KIM-1 were among the significantly regulated ones observed short-time after injection of [^177^Lu]Lu-DOTA-(Tyr^3^)-octreotate^18,21^. We also observed a dose- and time-related correlation between urinary RBP4 in mice and [^177^Lu]Lu-DOTA-(Tyr^3^)-octreotate exposure^22^. Additional proposed biomarkers were cyclin-dependent kinase inhibitor 1 A (CDKN1A), S100 calcium binding protein A6 (S100A6) and adiponectin (ADIPOQ). The use of all of these biomarkers was only studied relatively early after radiation exposure. Thus, there is a need to observe their expressions also late after exposure.

A short description of biomarkers used in this study are given as follows. Creatinine is produced by muscles, with a fairly constant production rate and is filtered by the kidneys. Creatinine level is commonly used clinically as a marker for kidney function. Under normal physiological conditions, the urinary creatinine level is relatively constant. It is therefore also commonly used to normalize urinary levels of other proteins to compensate for, e.g., diurnal variations in urine concentration^23^. Cystatin C is produced by nucleated cells and is also filtered in the kidney, but unlike creatinine most of the cystatin C is reabsorbed and degraded in proximal tubules^24^. NGAL is one of the most promising biomarkers for kidney damage with rapid upregulation and secretion from the kidney epithelium early after damage. It is filtered in glomerulus and reabsorbed in proximal tubules; increased urinary NGAL is a sign of kidney cell damage and impaired reabsorption in the proximal tubules will further elevate urinary NGAL^25^. KIM-1 is normally expressed in kidney tubule and has been associated with maintaining and repairing the tubule epithelium. Elevated urinary KIM-1 is an indicator of kidney damage and may also be a sign of cell repair^26^. KIM-1 is one of seven urinary proteins that were qualified as highly sensitive and specific urinary biomarkers to monitor drug-induced kidney injury in preclinical studies and on a case-by-case basis in clinical trials by the Food and Drug Administration (FDA)^27^. RBP4 acts as a vitamin A transporter in blood and is freely filtered in glomerulus and almost completely reabsorbed in proximal tubules. Under normal physiological conditions, the urinary RBP4 concentration is very low, but upon proximal tubular dysfunction elevated urinary RBP4 can be observed^28^. CDKN1A, also known as p21, is involved in cell cycle progression and is one of the top candidates of radiation-responsive biomarkers^29–31^. S100A6 is a calcium-binding protein and up-regulation of S100A6 has previously been associated with acute renal damage in mice^32^. ADIPOQ is a hormone with anti-inflammatory and insulin-sensitizing properties and is secreted from adipocytes. ADIPOQ has been suggested to play a part in the maintenance of normal kidney function, and elevated urinary and serum ADIPOQ levels have been associated with risk of chronic kidney disease^33,34^.

The aims of this study were to (1) evaluate the use of urinary RBP4, NGAL, creatinine and cystatin C as early responding biomarkers for [^177^Lu]Lu-DOTA-(Tyr^3^)-octreotate induced kidney damage in normal mice injected with [^177^Lu]Lu-DOTA-(Tyr^3^)-octreotate, and (2) to study the expression of NGAL, KIM-1, CDKN1A, S100A6 and ADIPOQ in kidney tissue from these mice to validate the usefulness of these proteins as indicators of toxicity. A third aim was to evaluate the long-term effect on morphology in kidneys in mice treated with [^177^Lu]Lu-DOTA-(Tyr^3^)-octreotate with and without A1M or A1M alone, in order to further evaluate the proposed kidney protective abilities of A1M during [^177^Lu]Lu-DOTA-(Tyr^3^)-octreotate treatment.

## Materials and methods

### Radiopharmaceutical and A1M

Preparations of radiopharmaceutical and A1M were conducted according to previously published method^35^. In short, octreotate was radiolabeled with ^177^Lu-chloride (LuMark^®^, Nuclear Research and Consultancy Group, IDB Holland, Netherlands) according to the manufacturer’s instructions, resulting in a specific activity of 37 MBq/µg. The ^177^Lu activity in each syringe was measured by a well-type ionization chamber CRC-15R, Capintec, Ramsey, New Jersey, USA) before and after injection to determine the amount of [^177^Lu]Lu-DOTA-(Tyr^3^)-octreotate administered to each mouse. Human recombinant A1M (modified variant A1M-035^36^ were supplied by A1M Pharma (Lund, Sweden) together with vehicle buffer (10 mM sodium phosphate buffer, 0.15 M CaCl, 2 mg/ml L-histidine, pH 7.4) that was used to dilute the A1M. A1M was dosed based on mouse body weight to a final dose of 5.0 mg/kg with A1M concentration of 0.77 mg/mL.

### Animal experiments and sample collection

Six-week-old female C57BL/6 N mice (Charles River Laboratories International, Salzfeld, Germany) were divided into 6 groups (*n* = 5/group) and *i.v.* injected in the tail vein with (1) phosphate buffered saline solution (PBS), (2) A1M, (3) 90 MBq [^177^Lu]Lu-DOTA-(Tyr^3^)-octreotate, (4) 150 MBq [^177^Lu]Lu-DOTA-(Tyr^3^)-octreotate, (5) 90 MBq [^177^Lu]Lu-DOTA-(Tyr^3^)-octreotate +A1M or (6) 150 MBq [^177^Lu]Lu-DOTA-(Tyr^3^)-octreotate+A1M. Injection with [^177^Lu]Lu-DOTA-(Tyr^3^)-octreotate was given first, followed about 1–2 min later by injection of A1M. To give an equal number of injections per animal, one additional PBS injection was given to all mice that had not already received two injections. The injections were given slowly and the total volume administered to each animal was 0.23–0.26 mL

Individual samples of urine were collected 1–2 days before injections to establish baseline (normal level) of investigated proteins. Urine was subsequently collected at about 1 week (6 days), one month (35 days), 4–5 months (131 days) and 7–8 months (223 days) after injection from the animals that received 150 MBq [^177^Lu]Lu-DOTA-(Tyr^3^)-octreotate, [^177^Lu]Lu-DOTA-(Tyr^3^)-octreotate+A1M, A1M or PBS. Sample collection was mainly performed in the morning. Individual mice were then kept in an empty cage for a short time (max 10 min). The urine was collected from the bottom of the cage with a pipette, flash frozen in liquid nitrogen and stored at -80 °C.

Nine months after injections, the experiment was terminated; the animals were euthanized with 0.5 ml of 60 mg/ml sodium pentobarbital (APL, Stockholm, Sweden) and then sacrificed by cardiac puncture. Kidneys were collected, fixed in 4% formaldehyde, later embedded in paraffin, and then prepared for immunohistochemical analysis as described below. According to previously published data, injection of 90 MBq and 150 MBq [^177^Lu]Lu-DOTA-(Tyr^3^)-octreotate would result in an absorbed dose of 40 and 54 Gy to the kidneys (extrapolated to infinity), respectively^37^.

All animals had free access to water, food and nesting materials. During the study, the mice were weighed about every second week and visual inspection of the general condition of the mice was performed every day. All animal procedures were approved by the Ethics Committee for Animal Research in Gothenburg, Sweden (ID 146–2015). All experiments were performed in accordance with relevant guidelines and are reported following guidelines from Animal Research Reporting of In Vivo Experiments (ARRIVE).

### Immunohistochemistry

Formalin fixed paraffin embedded (FFPE) kidney samples from mice sacrificed at 9 months after injection were sectioned at 2 µm. Sections were dried for one hour in 60°C before pre-treatment using DAKO PTLink system (Agilent Technologies, Santa Clara, California, USA) for deparaffinization and antigen retrieval with EnVision Flex target retrieval solution (Agilent Technologies, Santa Clara, Californian, USA) at pH 9. Sections were stained with hematoxylin and eosin. Immunohistochemical staining was performed using DAKO Autostainer Plus (Agilent Technologies) with EnVision peroxidase blocking reagent (Agilent Technologies), followed by applying the primary antibodies: S100A6 (ab181975, antibody concentration of 1:250), CDKN1A (ab188224, 1:500), ADIPOQ (ab22554, 1:500), NGAL (ab70287, 1:500) and KIM-1 (ab01032-23.0, 1:250) (all from Abcam, Cambridge, UK). Thereafter, FLEX/HRP (Agilent Technologies) was applied, and the tissues stained with DAB (3,3’-diaminobenzidine) (Agilent Technologies) and counterstained with EnVision FLEX hematoxylin (Agilent Technologies). Samples were washed with EnVision FLEX wash buffer (1x) (Agilent Technologies) between every step in the staining process and finally rinsed with deionized water. Dehydration was performed in increasing concentrations of ethanol (75, 95 and 99%) before cleared twice in xylene and coverslips were mounted with Pertex^®^ mounting medium for light microscopy (Histolab Products AB, Askim, Sweden).

Stained glasses were scanned with Leica SCN 400 (Leica microsystems, Wetzlar, Germany) and processed through the Leica image viewer software system. The evaluation of kidney histology and immunohistochemical analyses were performed by a kidney pathologist in a blinded procedure.

### Analysis of urinary biomarkers

The urine samples were thawed and then directly centrifuged to remove potential precipitates. Urinary biomarkers were analyzed in the groups that received injection of A1M, PBS, 150 MBq [^177^Lu]Lu-DOTA-(Tyr^3^)-octreotate or 150 MBq [^177^Lu]Lu-DOTA-(Tyr^3^)-octreotate+A1M. Mouse SimpleStep ELISA^®^ Kits from Abcam (Cambridge, UK) was used to measure concentration of cystatin C (ab201280), NGAL (ab199083) and RBP4 (ab202404) in collected urine samples from 4 of the 5 mice in these groups. Assay procedure and urine dilution were performed according to the manufacturer’s instructions. Optical density (OD) was measured at 450 nm using Perkin Elmer Victor 3 1420 Multilabel Plate Counter (Waltham, Massachusetts, USA). Samples with OD outside the range of the standard curve, were re-diluted and re-analyzed until an appropriate OD (within the standard curve) was achieved. Accordingly, the following dilution range were used for respective urine protein analysis: cystatin C 1:300-1:2000, NGAL 1:250-1:12000 and RBP4 1:15 − 1:1500. Concentration of creatinine was measured in urine (diluted 1:10 − 1:20 according to recommendation from manufacturer) using Creatinine assay from R&D Systems (Europe Ltd., Abingdon, UK). OD was measured as described above, but at 490 nm.

### Statistical analyses

Data on body weight is presented as mean values, and error bars indicate SEM. IBM SPSS Statistics (version 25) linear mixed model was used on the repeated weight measures to determine if there were differences in growth of the mice between the groups. Time and injected substances were chosen as fixed factors and the mouse number as random factor. If statistically significant difference of *p* ≤ 0.05 was obtained for time and injected substance, the mixed model analysis was followed by pairwise comparisons to determine which groups were statistically significant different from each other in total mean body weight (Bonferroni adjusted *p* ≤ 0.05). For the last time-point, IBM SPSS Statistics (version 25) independent t-test was used to determine which groups were statistically significant different from each other at that time point.

Data on urinary protein concentration was given as median values, and error bars indicate SEM. The protein concentration values were skewed and therefore log_10_-transformed before statistical analysis. The different biomarkers were analyzed separately in a sequential manner. First, IBM SPSS Statistics (version 25) linear mixed model was used on the repeated measures to determine if there were differences between any of the time-points and any of the groups. Time and injected substances were chosen as fixed factors and the mouse number as random factor. If statistically significant difference of *p* ≤ 0.05 was obtained for time and injected substance, the mixed model analysis was followed by pairwise comparisons to determine which groups were statistically significant different from each other (Bonferroni adjusted *p* ≤ 0.05). Last, IBM SPSS Statistics (version 25) independent t-test was used to determine at which time-point the groups were statistically significant different (*p* ≤ 0.05).

## Results

**Fig. 1:**
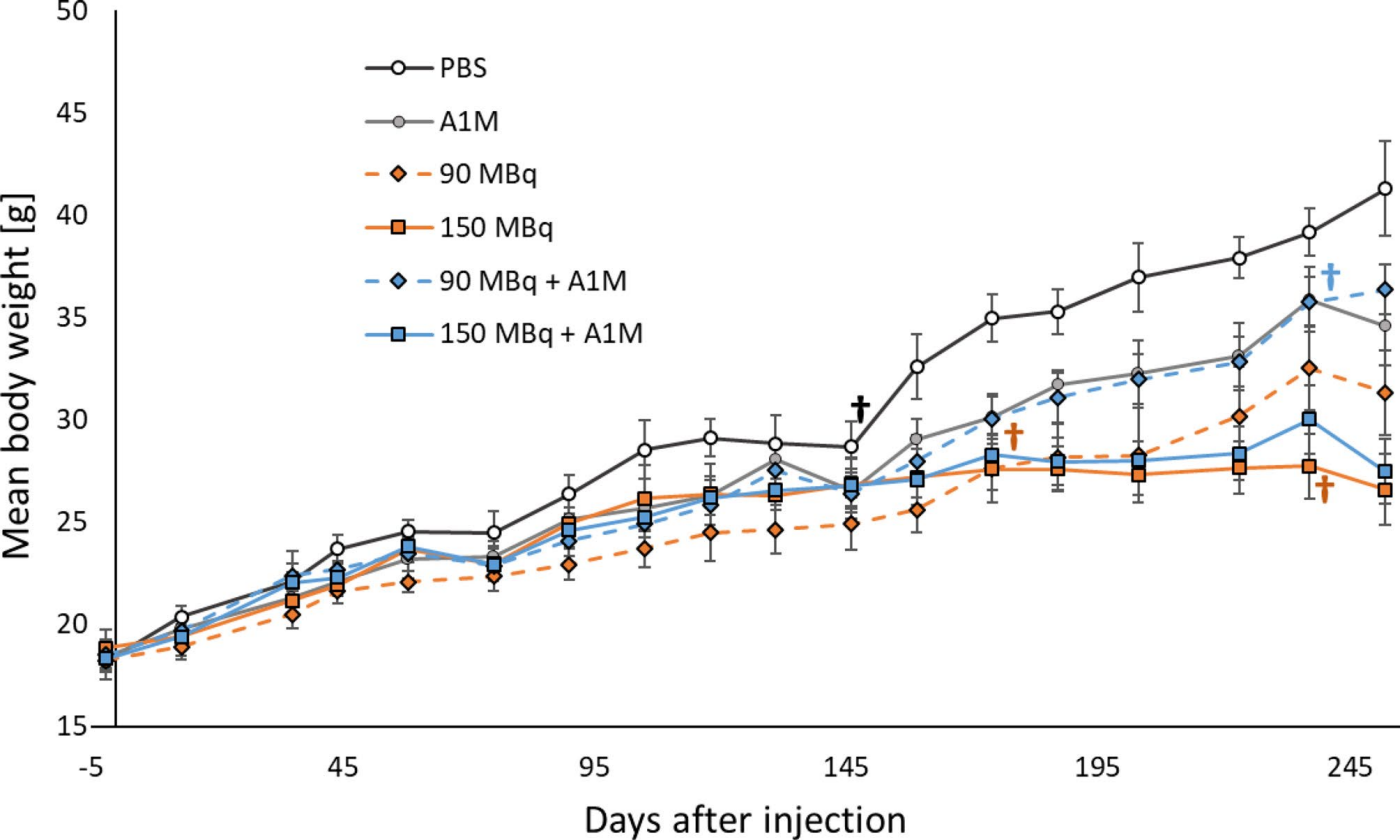
Body weight of the mice during study time. Female C57BL/6 N mice, aged 6 weeks at time of injection of PBS (white circles), A1M (gray circles), 90 MBq [^177^Lu]Lu-DOTA-(Tyr3)-octreotate (orange diamonds), 150 MBq [^177^Lu]Lu-DOTA-(Tyr3)-octreotate (orange squares), 90 MBq [^177^Lu]Lu-DOTA-(Tyr3)-octreotate + A1M (blue diamonds) or 150 MBq [^177^Lu]Lu-DOTA-(Tyr3)-octreotate + A1M (blue squares). Data is given as mean values (*n* = 5/group), and error bars indicate SEM. Four mice were sacrificed prior to end of the study, indicated by **†**.

During the study time, a total increase in body weight was observed with time for all mice (Fig. 1). The general condition of the mice was well with a few exceptions. Four mice were sacrificed prior to the end of the study; three of them due to stress symptoms: one from the 90 MBq [^177^Lu]Lu-DOTA-(Tyr^3^)-octreotate + A1M group at day 240 (no abnormality observed at postmortem autopsy), one from the 150 MBq [^177^Lu]Lu-DOTA-(Tyr^3^)-octreotate group at day 240 (had enlarged and discolored kidneys) and one from the PBS group at day 146 (no abnormality). The fourth mouse belonged to the 90 MBq [^177^Lu]Lu-DOTA-(Tyr^3^)-octreotate group and was sacrificed after 177 days due to reduced general condition, and postmortem autopsy indicated small-intestinal bleeding.

Differences in mean weight were observed between some of the groups (Table 1). The mice in the control groups (A1M and PBS group) had statistically significant higher total mean body weight than all other groups, except for A1M compared to 90 MBq [^177^Lu]Lu-DOTA-(Tyr^3^)-octreotate + A1M (mean difference of 0.1 g, p-value = 1.000). At the end of the study the PBS group had statistically significant higher mean weight than the other groups except for the 90 MBq [^177^Lu]Lu-DOTA-(Tyr^3^)-octreotate + A1M and A1M group. No statistically significant difference was observed between the two activity levels (150 MBq vs. 90 MBq: total mean difference of 0.2 g, p-value = 1.000 and mean difference at end of study of -4.7 g, p-value = 0.131). The mean body weight in the 150 MBq [^177^Lu]Lu-DOTA-(Tyr^3^)-octreotate group was not statistically significant different from corresponding combination group (150 MBq vs. 150 MBq + AM1: total mean difference of -0.2 g, p-value = 1.000 and mean difference at end of study of -0.88 g, p-value = 0.721). The total mean body weight in the 90 MBq [^177^Lu]Lu-DOTA-(Tyr^3^)-octreotate group was on the other hand statistically significant lower than in the corresponding combination group (90 MBq vs. 90 MBq + AM1: total mean difference of -2.0 g, p-value = 0.000) although this difference was not observed at the end of the study (90 MBq vs. 90 MBq + AM1: mean difference at the end of the study of -5.1 g, p-value = 0.139).

**Table 1:** Overview of the body weight of the mice in the different treatment groups. Data is given as difference in total mean weight (*n* = 5/group) between the groups and difference in mean weight (*n* = 4–5/group) at the end of study. SD is standard diviation and statistically significant difference between the groups are indicated as *.

(I) Treatment	(J) Treatment	Difference in total mean weight (I-J) [g]	SD	p-value	Difference in mean weight [g] (I-J) at end of study	SD	p-value
150 MBq ^177^Lu-oct	150 MBq ^177^Lu-oct + A1M	-0.23	0.42	1.000	-0.88	2.7	0.721
	90 MBq ^177^Lu-oct	0.17	0.43	1.000	-4.7	2.9	0.131
	90 MBq ^177^Lu-oct + A1M	-1.8*	0.42	0.000	-9.8*	2.9	0.012
	A1M	-2.0*	0.42	0.000	-8.0*	2.7	0.020
	PBS	-4.3*	0.43	0.000	-15*	2.9	0.002
150 MBq ^177^Lu-oct + A1M	150 MBq ^177^Lu-oct	0.23	0.42	1.000	0.88	2.7	0.721
	90 MBq ^177^Lu-oct	0.40	0.43	1.000	-3.8	2.7	0.174
	90 MBq ^177^Lu-oct + A1M	-1.6*	0.42	0.003	-8.9*	2.7	0.011
	A1M	-1.7*	0.42	0.001	-7.1*	2.6	0.021
	PBS	-4.1*	0.43	0.000	-14*	2.7	0.001
90 MBq ^177^Lu-oct	150 MBq ^177^Lu-oct	-0.17	0.43	1.000	4.7	2.9	0.131
	150 MBq ^177^Lu-oct + A1M	-0.40	0.43	1.000	3.8	2.7	0.174
	90 MBq ^177^Lu-oct + A1M	-2.0*	0.43	0.000	-5.1	2.9	0.139
	A1M	-2.1*	0.43	0.000	-3.2	2.7	0.287
	PBS	-4.5*	0.43	0.000	-10*	2.9	0.018
90 MBq ^177^Lu-oct + A1M	150 MBq ^177^Lu-oct	1.8*	0.42	0.000	9.8*	2.9	0.012
	150 MBq ^177^Lu-oct + A1M	1.6*	0.42	0.003	8.9*	2.7	0.011
	90 MBq ^177^Lu-oct	2.0*	0.43	0.000	5.1	2.9	0.139
	A1M	-0.14	0.42	1.000	1.8	2.7	0.545
	PBS	-2.5*	0.43	0.000	-4.9	2.9	0.169
A1M	150 MBq ^177^Lu-oct	2.0*	0.42	0.000	8.0*	2.7	0.020
	150 MBq ^177^Lu-oct + A1M	1.7*	0.42	0.001	7.1*	2.6	0.021
	90 MBq ^177^Lu-oct	2.1*	0.43	0.000	3.2	2.7	0.287
	90 MBq ^177^Lu-oct + A1M	0.14	0.42	1.000	-1.8	2.7	0.545
	PBS	-2.4*	0.43	0.000	-6.7	2.7	0.057
PBS	150 MBq ^177^Lu-oct	4.3*	0.43	0.000	15*	2.9	0.002
	150 MBq ^177^Lu-oct + A1M	4.1*	0.43	0.000	14*	2.7	0.001
	90 MBq ^177^Lu-oct	4.5*	0.43	0.000	10*	2.9	0.018
	90 MBq ^177^Lu-oct + A1M	2.5*	0.43	0.000	4.9	2.9	0.169
	A1M	2.4*	0.43	0.000	6.7	2.7	0.057

### Urinary biomarkers for kidney damage

**Fig. 2:**
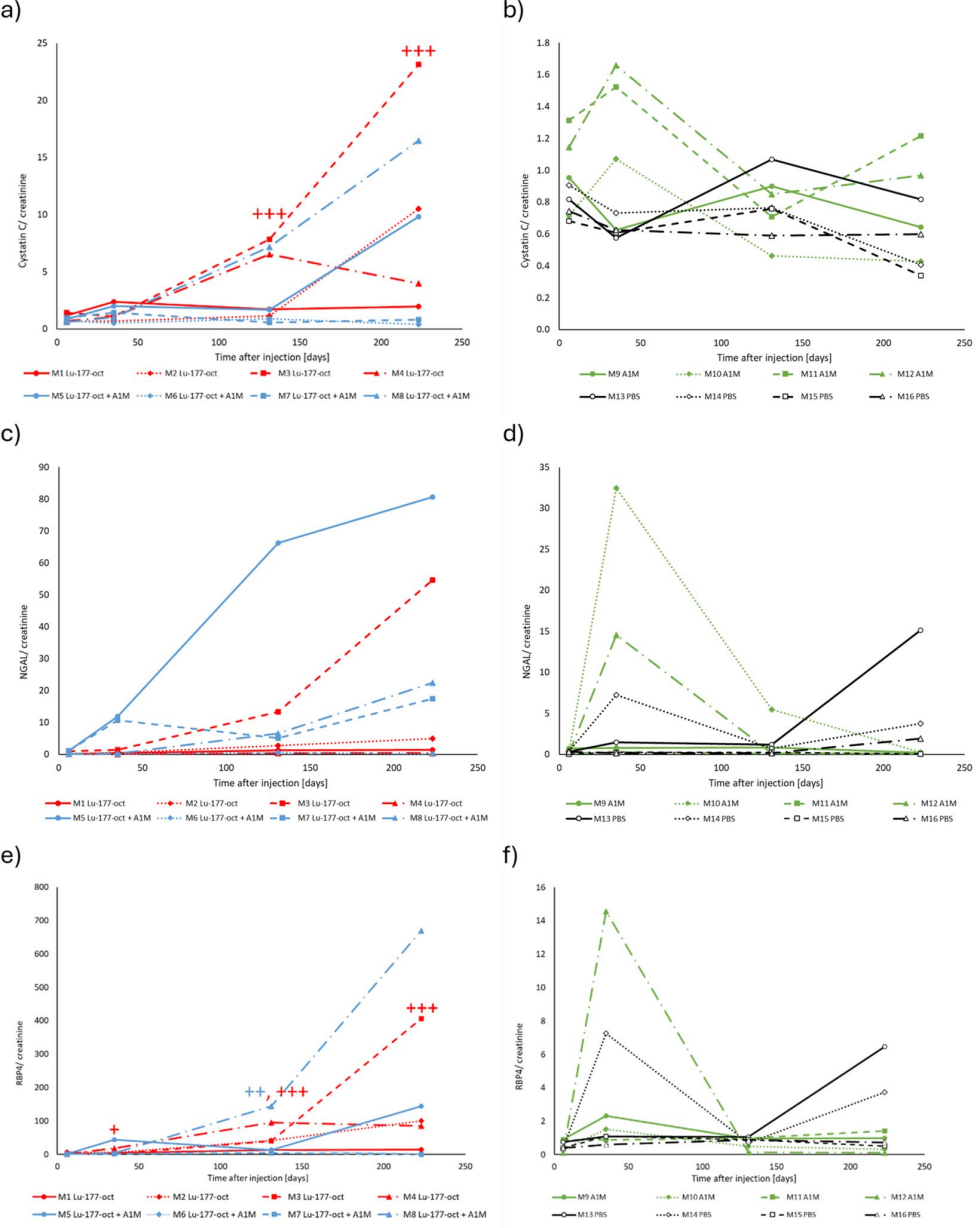
Levels of urinary biomarkers for kidney damage relative baseline and creatinine levels. Urinary concentration of (**a**-**b**) cystatin C/creatinine, (**c**-**d**) NGAL/creatinine, and e-f) RBP4/creatinine, versus time after injection from mice injected with 150 MBq [^177^Lu]Lu-DOTA-(Tyr3)-octreotate (orange), 150 MBq [^177^Lu]Lu-DOTA-(Tyr3)-octreotate + A1M (blue), A1M (gray) or PBS (white). All values are normalized against baseline (− 2 days). Statistical analysis were performed on log_10_-transformed data, and statistically significant difference between the groups at each time point are indicated in the figure as ^+^ the value is different from the value in the PBS group; ^++^ the value is different from the value in the A1M group, and ^+++^ the value is different from the values in both the A1M and PBS groups.

Concentrations of creatinine, cystatin C, NGAL and RBP4 measured in urine at baseline (-2 days), 6, 35, 131 and 223 days after injection of 150 MBq [^177^Lu]Lu-DOTA-(Tyr^3^)-octreotate, 150 MBq [^177^Lu]Lu-DOTA-(Tyr^3^)-octreotate+A1M, A1M or PBS are shown in Table 2. To compensate for the skewedness of the distribution of the protein levels, the values were log_10_-transformed before group comparison were made. To visualize relative change in urinary levels from baseline and to compensate for diurnal variation in urine concentration, the levels of cystatin C, NGAL and RBP4 were normalized against baseline and creatinine concentration (Fig. 2).

**Table 2:** Concentration of creatinine, cystatin C, NGAL and RBP4 in urine from mice at several time-points before and after injection of 150 MBq [^177^Lu]Lu-DOTA-(Tyr^3^)-octreotate, 150 MBq [^177^Lu]Lu-DOTA-(Tyr^3^)-octreotate+A1M, A1M or PBS. Data are given as median values with range in brackets. Statistical analyses were performed on log_10_-transformed data, and statistically significant differences between the groups at each time point are indicated as ^1^ different from PBS; ^2^ different from A1M; ^3^ different from both A1M and PBS.

Treatment	Time after injection [d]	Creatinine [mg/dl]		Cystatin C [ng/ml]		NGAL [ng/ml]		RBP4 [ng/ml]	
150 MBq ^177^Lu-oct	-2	37	(33–54)	290	(210–370)	160	(62–1300)	2.8	(1.2–4.5)
	6	56	(46–81)	390	(340–490)	44	(28–75)	7.6	(2.3–13)
	35	49	(33–77)	430	(310–590)	86	(30–90)	13^3^	(8.7–48)
	131	42^1^	(22–54)	970	(510–1300)	390	(340–470)	90^3^	(51–170)
	223	20^1^	(11–78)	960^1^	(340–1300)	360	(130–490)	110^3^	(35–260)
150 MBq ^177^Lu-oct + A1M	-2	46	(40–52)	410	(340 − 490)	160	(21–1,200)	4.6	(2.5–20)
	6	58	(42–91)	450	(220–620)	39	(15–180)	4.0	(2.0–5.8)
	35	39	(20–67)	350	(210–670)	71	(18–1900)	15^2^	(6.3–81)
	131	43^1^	(32–51)	480	(240–2,000)	900	(520–1,000)	71^3^	(46–285)
	223	12^1^	(6–23)	330	(100–930)	430	(50–940)	44	(1.4–260)
A1M	-2	44	(27–48)	270	(200–390)	100	(37–330)	2.1	(1.2–4.6)
	6	94	(84–98)	650	(570–700)	59	(38–91)	3.4	(2.8–3.8)
	35	41	(11–86)	270	(110–670)	280	(63–2,600)	2.6	(1.5–6.0)
	131	63	(46–80)	300	(290–320)	55	(32–470)	3.5	(2.0–3.7)
	223	51	(38–73)	290	(171–370)	23	(14–27)	3.0	(1.5–3.8)
PBS	-2	46	(42–54)	400	(380–480)	140	(61–220)	2.5	(2.4–3.6)
	6	66	(65–97)	480	(430–650)	29	(23–100)	2.3	(2.1–2.3)
	35	47	(32–72)	260	(190–400)	66	(40–470)	2.5	(2.1–3.6)
	131	84	(68–88)	580	(360–810)	96	(60–110)	4.5	(3.1–5.0)
	223	81	(50–93)	330	(240–500)	614	(26–860)	4.4	(2.8–15)

No statistically significant differences in urinary creatinine were observed between any of the groups up to day 35 after injection (Table 1). After 131 and 223 days, the creatinine levels decreased in the [^177^Lu]Lu-DOTA-(Tyr^3^)-octreotate (median value 42 mg/dl at 131 days and 20 mg/dl at 223 days) and [^177^Lu]Lu-DOTA-(Tyr^3^)-octreotate+A1M (median value 43 mg/dl at 131 days and 12 mg/dl at 223 days) groups and were statistically significant lower than in the PBS control group (131 days: median value 84 mg/dl, *p* = 0.033 vs. ^177^Lu, and *p* = 0.002 vs. ^177^Lu+A1M, 223 days: median value 81 mg/dl, *p* = 0.016 vs. ^177^Lu, and *p* = 0.006 vs. ^177^Lu+A1M).

No significant change was observed in urinary cystatin C level in any of the groups until day 223 (Table 2). Then, the cystatin C level had increased in [^177^Lu]Lu-DOTA-(Tyr^3^)-octreotate group (median value 960 ng/ml) and was statistically significant higher than in PBS control group (median value 330 ng/ml, *p* = 0.039). No differences were observed between any of the other groups. When normalized against baseline and creatinine, cystatin C levels at days 131 and 223 after [^177^Lu]Lu-DOTA-(Tyr^3^)-octreotate injection were statistically significant higher (median value 4.1 and 7.2) than in both A1M (median value 0.8, *p* = 0.025 at day 131 and median value 0.8, *p* = 0.001 at day 223) and PBS controls (median value 0.8, *p* = 0.030 at day 131 and median value 0.5, *p* = 0.013 at day 223) (Fig. 2a). At day 223, an increase was also observed in the combination group (median value 5.3), although not statistically significant.

The median urinary NGAL concentration differed over time for all groups (Table 2). In the combination group, a trend of increase in NGAL level was observed relative baseline and creatinine levels (Fig. 2b). The linear mixed model analyses showed a statistically significant difference in the absolute and relative concentration of NGAL with time (*p* = 0.001). Since no difference was found for the factor “injected substance” (*p* = 0.154), no pairwise comparison between groups were made.

The urinary RBP4 concentration from mice injected with A1M or PBS remained at baseline-level throughout the study time (Table 2). In the irradiated groups, the urinary RBP4 levels increased with time. At 35 days after injection of [^177^Lu]Lu-DOTA-(Tyr^3^)-octreotate with or without A1M (median value 15 and 13 ng/ml), the levels were statistically significant higher than in the A1M group (median value 2.6 ng/ml, *p* = 0.039 vs. ^177^Lu+A1M and *p* = 0.011 vs. ^177^Lu). At this time-point, the RBP4 level in the [^177^Lu]Lu-DOTA-(Tyr^3^)-octreotate group was also significantly higher than in the PBS group (median value 2.5 ng/ml, *p* = 0.004). The increased urinary RBP4 level in the ^177^Lu-octreotate group remained until the end of the study (median value 90 ng/ml at day 131 and 110 ng/ml at day 223) and were statistically significant higher than both A1M (median value 3.5 ng/ml, *p* = 0.000 at day 131 and 3.0 ng/ml, *p* = 0.004 at day 223) and PBS groups (median value 4.5 ng/ml, *p* = 0.000 at day 131 and 4.4 ng/ml, *p* = 0.005 at day 223). In the combination group, the RBP4 level was still elevated at day 131 (median value 71 ng/ml), but at day 223 the median RBP4 level decreased again (median value 44 ng/ml) and was not different from the PBS or A1M groups. RBP4 levels increased with time after injection of [^177^Lu]Lu-DOTA-(Tyr3)-octreotate with or without A1M, also after normalization against baseline and creatinine levels (Fig. 2c). The decrease in median concentration of RBP4 in the combination group at the latest time-point was not seen in the normalized data (median 72). No statistically significant difference in urinary concentration (absolute or relative values) of any of the proteins was observed between [^177^Lu]Lu-DOTA-(Tyr3)-octreotate and [^177^Lu]Lu-DOTA-(Tyr3)-octreotate +A1M groups at any time-point. The same findings were also seen for the non-irradiated groups: A1M vs. PBS.

### Morphological signs of kidney injury

**Fig. 3:**
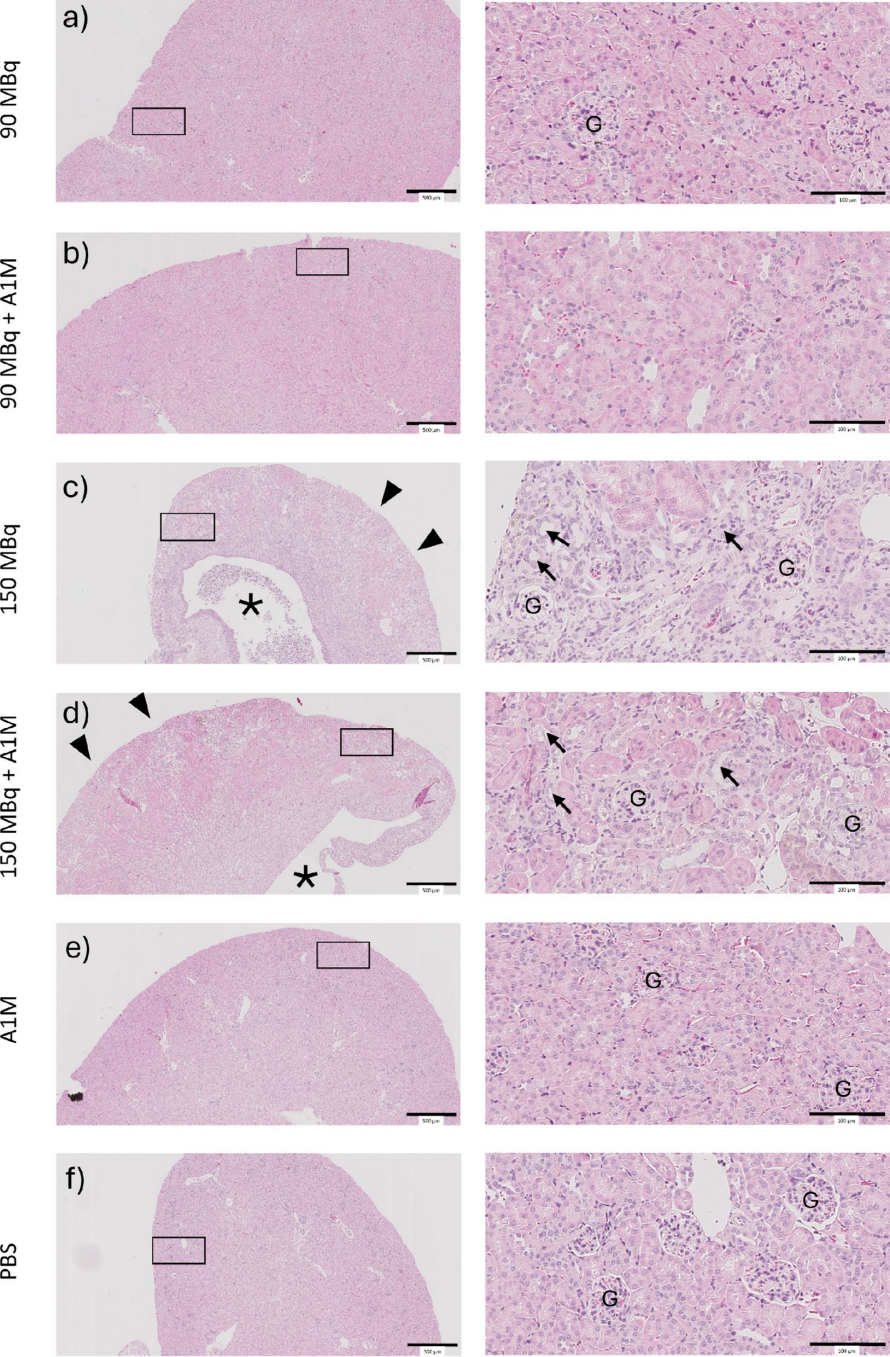
Kidney morphology of the investigated groups. Fig (**a**) shows kidney histology following exposure to 90 MBq [^177^Lu]Lu-DOTA-(Tyr3)-octreotate, whereas Fig (**b**) shows kidney histology after exposure to 90 MBq [^177^Lu]Lu-DOTA-(Tyr3)-octreotate in the presence of A1M. The morphology is without remark in both groups. No signs of fibrosis or tubular atrophy can be seen and the glomeruli are without changes. Figs (**c** and **d**) show the kidney tissue following exposure to 150 MBq [^177^Lu]Lu-DOTA-(Tyr3)-octreotate in the absence and presence of A1M, respectively. The histological changes are similar in both conditions. Macroscopically a dilation of the renal pelvis can be appreciated (asterisk). Also, a distinct reduction of the cortical thickness is evident also at lower magnification corresponding to tubular atrophy and fibrosis (arrowheads). This is shown in the inserts of higher magnification shown in highlighting the tubular atrophy (arrows) and interstitial fibrosis. A generalize edema is also seen in the tissue. In Figs (**e** and **f**), kidney tissue is shown from the groups receiving only A1M or PBS. No signs of tubular or glomerular histological changes were seen in these groups. Staining by hematoxylin-eosin. Increased magnifications of areas in boxes are shown in the right column of images. Scale bar 500 μm in left column and 100 μm in right column. G= glomerulus.

Representative morphological images of kidneys from each group are presented in Fig. 3. The groups that were exposed to 90 MBq [^177^Lu]Lu-DOTA-(Tyr3)-octreotate and 90 MBq [^177^Lu]Lu-DOTA-(Tyr3)-octreotate +A1M displayed a similar morphology. No signs of tubular or glomerular injury were seen in any of the groups (Figs. 3a-b). The groups receiving 150 MBq ^177^Lu-octreotate and 150 MBq [^177^Lu]Lu-DOTA-(Tyr3)-octreotate +A1M showed advanced tubular injury mainly affecting the tubular compartment. The glomerular morphology was unremarkable, but the cortical as well as medullary tubular segments were severely injured with on average 75% tubular atrophy and fibrosis (*n* = 4). Remaining tubules focally showed signs of subtotal injury in the form of tubular simplification and dilation. No improvement in these changes was seen in the group also given A1M, which also showed similar degree of fibrosis (*n* = 4) (Figs. 3c-d). The non-irradiated groups (PBS and PBS+A1M) showed no histological changes (Figs. 3e-f).

### Immunohistochemical staining for biomarkers for kidney damage

**Fig. 4:**
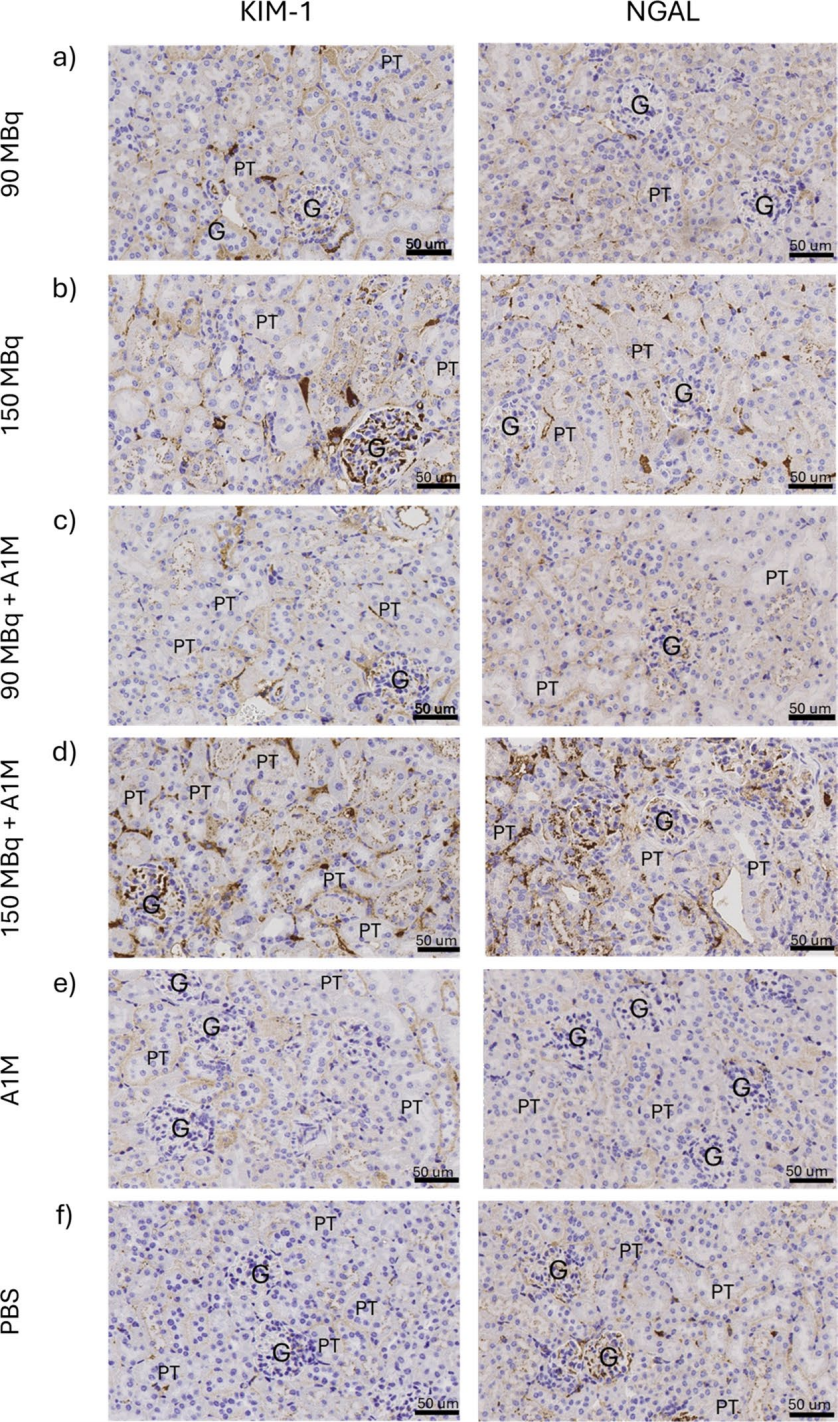
Immunohistochemical staining for KIM-1 and NGAL in mouse kidney. Fig (**a**), left column, shows a very slight apical KIM-1 expression in about 30% of tubules and right column shows endothelium expression of cytoplasmic NGAL staining after exposure to 90 MBq [^177^Lu]Lu-DOTA-(Tyr3)-octreotate. A similar location and expression of KIM-1 and NGAL is seen following exposure to 90 MBq [^177^Lu]Lu-DOTA-(Tyr3)-octreotate in the presence of A1M (Fig **b**). Fig (**c**), left column, shows apical expression of KIM-1 in about 50% of tubules and right column shows about cytoplasmic expression of NGAL in 25% of proximal tubules in the 150 MBq [^177^Lu]Lu-DOTA-(Tyr3)-octreotate group. Similar KIM-1 and NGAL expressions are shown in the 150 MBq [^177^Lu]Lu-DOTA-(Tyr3)-octreotate+ A1M group (Fig **d**). In Fig (**e**) the KIM-1 and NGAL expressions are shown from the groups receiving only A1M or PBS. NGAL is only expressed in the endothelium and low apical expression of KIM-1 is observed. Tissues were counterstained with hematoxylin after primary antibody staining. Scale = 50 μm, G= glomerulus and PT= proximal tubules.

Expression of KIM-1 and NGAL was observed in kidney tissue (Fig. 4; Table 3). A dose-dependent expression of KIM-1 was observed with a moderate tubular expression in the 90 MBq [^177^Lu]Lu-DOTA-(Tyr3)-octreotate group (Fig. 4a) and increased expression in the 150 MBq [^177^Lu]Lu-DOTA-(Tyr3)-octreotate group (Fig. 4b). No elevated expression of NGAL was observed in the 90 MBq [^177^Lu]Lu-DOTA-(Tyr3)-octreotate group compared with the PBS group (Figs. 4a, f), but exposure to 150 MBq resulted in increased tubular NGAL expression (Fig. 4b). The expression of KIM-1 and NGAL in the combination groups ([^177^Lu]Lu-DOTA-(Tyr3)-octreotate+A1M) was not different from corresponding [^177^Lu]Lu-DOTA-(Tyr3)-octreotate groups at any activity level (Figs. 4a-b, c-d). Exposure to A1M alone did not alter the expression of KIM-1 or NGAL (Fig. 4e) compared to the PBS groups (Fig. 4f).

**Fig. 5:**
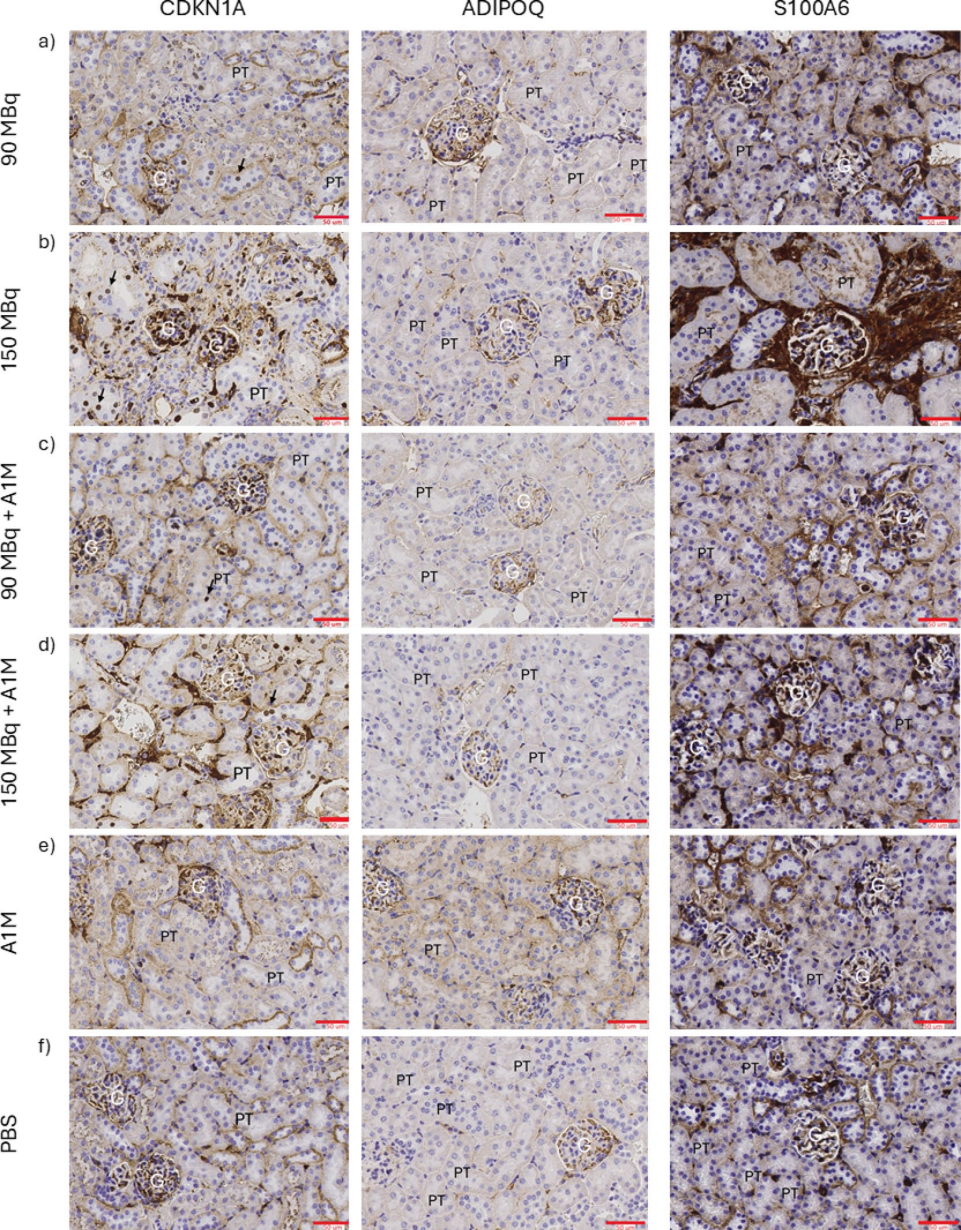
Immunohistochemical staining for CDKN1A, ADIPOQ and S100A6 in mouse kidney. Fig (**a**) shows moderate expression of CDKN1A (see arrow), low focal endothelial expression of ADIPOQ, and expression of S100A6 in about 20% of tubules after exposure to 90 MBq [^177^Lu]Lu-DOTA-(Tyr3)-octreotate. Fig (**b)** shows a high expression of CDKN1A (see arrow), moderate expression of ADIPOQ and expression of S100A6 in about 60% of tubules after exposure to 150 MBq [^177^Lu]Lu-DOTA-(Tyr3)-octreotate. The expression of these proteins following exposure to 90 MBq [^177^Lu]Lu-DOTA-(Tyr3)-octreotate did not change in the presence of A1M (Fig **c**). The expression of CDKN1A (see arrow) and ADIPOQ was not changed when 150 MBq [^177^Lu]Lu-DOTA-(Tyr3)-octreotate were combined with A1M (Fig d, left and middle column). A slightly less prominent expression of S100A6 was found when 150 MBq [^177^Lu]Lu-DOTA-(Tyr3)-octreotate were combined with A1M (Fig **d**, right column). A low expression of CDKN1A, ADIPOQ and S100A6 are shown after injection of A1M or PBS (Fig **e**-**f**). Tissues were counterstained with hematoxylin after primary antibody staining. Scale bars = 50 μm, G= glomerulus and PT= proximal tubules.

Expression of CDKN1A, ADIPOQ and S100A6 was observed in kidney tissue (Fig. 5; Table 3). A dose-dependent increase in tubular expression of CDKN1A was observed: moderate expression in the 90 MBq, high expression in the 150 MBq [^177^Lu]Lu-DOTA-(Tyr3)-octreotate group and no expression in the PBS and A1M groups (Figs. 5a-b, e-f). The [^177^Lu]Lu-DOTA-(Tyr3)-octreotate induced CDKN1A expression was in general similar to that in the [^177^Lu]Lu-DOTA-(Tyr3)-octreotate+A1M groups (Figs. 5a-b, c-d). Focal endothelial expression of ADIPOQ was observed in kidneys from mice exposed to 90 MBq [^177^Lu]Lu-DOTA-(Tyr3)-octreotate or 90 MBq [^177^Lu]Lu-DOTA-(Tyr3)-octreotate+A1M, PBS and A1M (Figs. 5a, c,e-f). Moderate ADIPOQ expression (about 20%) was observed in the interstitial and proximal tubules cells in both the 150 MBq [^177^Lu]Lu-DOTA-(Tyr3)-octreotate and the 150 MBq [^177^Lu]Lu-DOTA-(Tyr3)-octreotate+A1M groups (Figs. 5b, d). Dose-dependent increase in S100A6 expression with moderate expression for mice exposed to 90 MBq and further higher expression in the 150 MBq [^177^Lu]Lu-DOTA-(Tyr3)-octreotate group was observed (Figs. 5a-b). Co-administration of A1M had no effect in the 90 MBq group but resulted in slightly less S100A6 expression in the 150 MBq [^177^Lu]Lu-DOTA-(Tyr3)-octreotate group (Figs. 5c-d). Low expression of S100A6 was observed along the tubules and proximal tubular cells in non-irradiated mice (Figs. 5e-f).

**Table 3:** Morphological evaluation of potential biomarkers for kidney damage. Results of morphological evaluation of expression of ADIPOQ, CDKN1A, KIM-1, NGAL and S100A6 in mouse kidney 9 months after injection of 90 MBq [^177^Lu]Lu-DOTA-(Tyr3)-octreotate with or without A1M, 150 MBq [^177^Lu]Lu-DOTA-(Tyr3)-octreotate with or without A1M, A1M only or PBS (control).

	ADIPOQ	CDKN1A*	KIM-1	NGAL	S100A6
90 MBq ^177^Lu- oct	focal endothelial expression	++	20% expression	endothelium only	20% along tubules, also proximal tubule cells
	focal endothelial expression	++	20-% expression	endothelium only	25% long tubules and focally strong expression
	focal endothelial expression, enhanced in medulla	+	no expression	endothelium only	20% along tubules, also proximal tubule cells
	focal endothelial expression	+	no expression	endothelium only	10% along tubules, also proximal tubule cells
90 MBq ^177^Lu- oct + A1M	focal endothelial expression	++	no expression	endothelium only	20% along tubules, also proximal tubule cells
	strong focal endothelial expression in fibrotic areas	++	about 50% expression in tubules	endothelium expression, about 40% of tubules express apically	80% of tubuli and focally strong expression
	focal endothelial expression	+	slight expression in 30% of tubules	endothelium only, enhanced in medulla	20% along tubules, also proximal tubule cells
	focal endothelial expression	+	slight expression in 30% of tubules	endothelium only	15% along tubules, also proximal tubule cells
150 MBq ^177^Lu- oct	focal endothelial expression	+++	50% expression	25% expression in proximal tubules	20% along tubules, also proximal tubule cells
	20% in interstitium, also in proximal tubule cells	++++	50–60% expression	50% apical expression	80% coverage
	30% expression in interstitium	++++	60% expression	25% tubular and enhanced in endothelium	60% coverage
	focal endothelial expression	+	no expression	endothelium only	20% along tubules, also proximal tubule cells
150 MBq ^177^Lu- oct + A1M	Focal expression in stroma, does not reflect degree of injury	+++	40–50% expression	60% of tubular cells show apical positivity	25% of the cortex
	focal endothelial expression	++	faint expression in 20%	endothelium only	20% along tubules, also proximal tubule cells
	focal endothelial expression	++	30% expression	endothelium only	20% dense in interstitium
	diffuse expression in interstitium and injured tubules	+++	50% expression, however severe distortion	30%, but hard to estimate due to advanced destruction	cannot be assessed due to fallout
	20% in interstitium, also in proximal tubule cells	+++	80% expression	40% apical expression	80% coverage
A1M	focal endothelial expression	0	10% expression	endothelium only	10% along tubules, also proximal tubule cells
	focal endothelial expression	0	15% expression	endothelium only	10% along tubules, also proximal tubule cells
	focal endothelial expression	0	5% expression	endothelium only	10% along tubules, also proximal tubule cells
	focal endothelial expression	0	30% expression	endothelium only	10% along tubules, also proximal tubule cells
PBS	focal endothelial expression	+	15% expression	endothelium only	10% along tubules, also proximal tubule cells
	focal endothelial expression	0	no expression	endothelium only	10% along tubules, also proximal tubule cells
	focal endothelial expression	0	no expression	endothelium only	10% along tubules, also proximal tubule cells
	focal endothelial expression	0	5% expression	endothelium only	10% along tubules, also proximal tubule cells

*scale 0-++++, where ++++ is extremely high expression.

## Discussion

In this study we evaluated the possibility to predict late [^177^Lu]Lu-DOTA-(Tyr3)-octreotate induced kidney damage by the use of proposed urinary biomarkers and correlate the data with histological and immunohistochemical analyses of kidney tissue. Urinary RBP4, NGAL, creatinine and cystatin C levels were measured at several time-points after injection of 150 MBq [^177^Lu]Lu-DOTA-(Tyr3)-octreotate. Kidney expressions of NGAL, KIM-1, CDKN1A, S100A6 and ADIPOQ were evaluated with immunohistochemical analyses 9 months after injection of 90 or 150 MBq [^177^Lu]Lu-DOTA-(Tyr3)-octreotate. These biomarkers, together with morphological evaluation of the kidneys, were used to detect any potential damage to the kidneys and to investigate if kidney injury could be prevented by co-administration of the proposed kidney protector A1M.

To be able to relate the long-term effects found in this study with short-term effects examined in previous studies, some parameters were kept the same, such as dose of A1M (5.0 mg/kg body weight) and the use of female C57BL/6 N mice. The strain used in this study differs from those applied in some of our previous studies on A1M, where female Balb/c nude mice were used for studies on tumor-bearing mice^35,38^. Sex and strain of the mice may to some extent influence the pharmacokinetics of [^177^Lu]Lu-DOTA-(Tyr3)-octreotate. These aspects were not investigated in the present study and should be addressed in future studies. However, based on our previous mouse experiments done with [^177^Lu]Lu-DOTA-(Tyr3)-octreotate, we expect such differences to be moderate, and should not influence the overall results and conclusions from these studies. It should also be noted that all results in this and in our previous studies were compared with corresponding data from controls in the same experiment, using the same type and batch of animals.

To be able to study the effect of a potential radioprotector we need to give high enough amounts of radioactivity that we know will have negative effects, and when studying long-term effects, the animals should also survive. This will better resemble the clinical situation. In the present study the activities injected were chosen based on previous experiments, where we detected negative effects on kidney function^21^. In that study we examined toxic effects in kidney 4, 8 and 12 months after administration of 30, 60, 90, 120, and 150 MBq [^177^Lu]Lu-DOTA-(Tyr3)-octreotate to mice. Morphological signs of toxicity were only found in kidneys 8 and 12 months after administration of 150 MBq. Injected activity of 90 and 150 MBq, in the present study, give an absorbed dose to kidneys of about 40 and 54 Gy^37^, which is higher than used in clinical routine (23–28 Gy). This was intentional since the purpose of the proposed procedure is to increase the number of treatment cycles and thus exceed the absorbed dose limit widely used today. Furthermore, clinical studies have shown that many patients without certain risk factors can receive a dose to the kidney up to at least 40 Gy without nephrotoxicity signs^3,4^. In the present study, kidney toxicity was demonstrated at these absorbed dose levels, indicating that the radiosensitivity of kidneys in mice might be higher than in humans. Another difference between mouse and human is a difference in absorbed dose distribution in kidneys between mice and man due to the short range of emitted electrons from ^177^Lu, where the mouse kidney would receive a more homogeneous dose distribution due to the smaller size. Thus, the kidney medulla in mice might have received a higher dose than in man. However, the morphological effects were mainly found in the proximal tubules in mouse kidney cortex. Thus, a direct translation from mouse to human regarding radiosensitivity is difficult, but the aims were to evaluate the usefulness of A1M and potential urinary and tissue biomarkers for radiation-induced effects. Especially the proposed urinary biomarkers should be tested in patients, to study dose-response relationships and to study the potential clinical use to predict late kidney toxicity.

To account for variations in the urine concentration due to e.g. variations in water consumption, the urinary protein concentration can be normalized against total urine volume. This was, however, not possible in this experimental setting. The urinary protein concentrations were instead normalized against the creatinine level. The time-points for urine collection were chosen based on our previous study, with similar setup, where effects on RBP4 were seen 30 days after injection^22^.

In the present study, the creatinine levels were relatively constant up to day 35 after injection of 150 MBq ^177^Lu-octreotate, but were lower at days 131 and 223. This finding could indicate reduced GFR in the irradiated mice. This assumption is strengthened by the findings in the cystatin C results, with increased levels in samples from both ^177^Lu-octreotate groups from day 131. Otherwise, besides the effect of kidney function, creatinine levels are also influenced primarily by production of creatine in the liver and production of creatinine in muscle tissue and thus physical activity. In the present study, we do not suspect any radiation-induced effects on liver and muscle tissues, and no differences in physical activity were generally observed between the groups. Therefore, together with the cystatin C data, we find it most possible to be an effect of reduced kidney function.

No consistent changes were observed in urinary NGAL, neither in irradiated nor non-irradiated mice. The immunohistochemical staining for NGAL showed evaluated tubular expression in kidneys from mice injected with 150 MBq [^177^Lu]Lu-DOTA-(Tyr3)-octreotate. These results indicate that NGAL can be upregulated in kidney tissue, 9 months after injection of 150 MBq [^177^Lu]Lu-DOTA-(Tyr3)-octreotate, and that urinary NGAL may be insufficient as early predictor of [^177^Lu]Lu-DOTA-(Tyr3)-octreotate induced kidney damage.

Among the urinary biomarkers investigated in this study, RBP4 showed the most prominent response to irradiation with increased concentration from day 35 after injection of 150 MBq [^177^Lu]Lu-DOTA-(Tyr3)-octreotate. Compared to the observed response of urinary cystatin C and NGAL, RBP4 was an early responding biomarker. These results are in agreement with our previous study on urinary RBP4 in [^177^Lu]Lu-DOTA-(Tyr3)-octreotate exposed mice^22^. In that study, a dose dependent increase of RBP4 levels was detected in mouse urine from 30 days after injection of 60 or 120 MBq [^177^Lu]Lu-DOTA-(Tyr3)-octreotate. No morphological changes were then observed in the kidneys at the end of that study (90 days after injection). In the present study morphological signs of kidney injury were found at a later time-point (9 months after injection) after injection of 150 MBq [^177^Lu]Lu-DOTA-(Tyr3)-octreotate. This finding is completely in line with the negative effects on kidney function demonstrated in a similar setting^21^. Taken together, these results advocate that RBP4 can be used as an early responding biomarker for [^177^Lu]Lu-DOTA-(Tyr3)-octreotate induced kidney damage. RBP4 can potentially be used to optimize the number of treatment cycles and can provide better options for individualizing the treatment.

The overall response in urinary levels of the investigated proteins did not change when [^177^Lu]Lu-DOTA-(Tyr3)-octreotate were co-injected with A1M. Even so, a trend of lower levels of urinary RBP4 and cystatin C was observed at later time-points in the [^177^Lu]Lu-DOTA-(Tyr3)-octreotate+A1M group compared to the [^177^Lu]Lu-DOTA-(Tyr3)-octreotate group. Furthermore, the RBP4 and cystatin C levels in the combination group was not statistically different from the controls to the same extent as the [^177^Lu]Lu-DOTA-(Tyr3)-octreotate only group. Since the statistical uncertainties were high for these data points (due to individual differences between mice within a group), it is difficult to draw any firm conclusions based on the observed trend. In a similar study on mice injected with 150 MBq [^177^Lu]Lu-DOTA-(Tyr3)-octreotate, the urinary albumin/creatinine value was lower in animals co-injected with A1M, indicating reduced general proteinuria^39^. Furthermore, the optimum dosage and timing of A1M administration in relation to the administered activity is still not established. We have previously investigated short-term effects of different administration schedules with up to five A1M injections (5 mg/kg) in combination with single injection of 60 MBq [^177^Lu]Lu-DOTA-(Tyr3)-octreotide^40^. No clear differences in concentration of RBP4 or creatinine in urine from mice 6–10 weeks after [^177^Lu]Lu-DOTA-(Tyr3)-octreotide injection were shown between the investigated administration schedules. However, it cannot be excluded that multiple administrations of A1M or higher dosage can have a radioprotective effect for higher activities and at later time-points.

The histological evaluation showed induced toxicity in kidneys from mice injected with 150 MBq [^177^Lu]Lu-DOTA-(Tyr3)-octreotate with or without A1M. Effects were observed in the tubular compartments with no significant effects on glomeruli. These findings are in agreement with previously observed [^177^Lu]Lu-DOTA-(Tyr3)-octreotate induced morphological changes in kidneys of rat and nude mice, showing greatest effect on the tubules^41–43^. However, we previously showed histological changes localized to the glomeruli 12 months after injection of 150 MBq [^177^Lu]Lu-DOTA-(Tyr3)-octreotate, while there were no signs of injury on tubules, interstitium and vasculature^20^.

To the best of our knowledge, only two clinical studies have been performed to investigate urinary and/or serum KIM-1 as biomarker for kidney damage from [^177^Lu]Lu-DOTA-(Tyr3)-octreotate treatment^44,45^. Bober et al. found that urinary and serum KIM-1 levels two days after injection of 7.4 GBq [^177^Lu]Lu-DOTA-(Tyr3)-octreotate were not significantly affected compared to baseline. Such a low activity will rarely result in any permanent kidney injury and could therefore not be predicted by KIM-1.1 mL/min/1.73m^2^ (SD = 32 mL/min/1.73m^2^) one year after treatment, the reduction is classified as mildly decreased (relative to young adult level)^46^. Both studies investigate activity levels within today’s standard treatment limit which seldom leads to any permanent kidney toxicity and can therefore not be predicted by KIM-1. The immunohistochemical staining for KIM-1 in the present study, revealed a dose dependent expression that advocates the use of KIM-1 as a biomarker for radiation-induced kidney damage. No urinary KIM-1 levels could be determined in this study, since the volume of urine available was limited. Thus, urinary and serum KIM-1 should be prioritized in similar future renal toxicity studies. Further studies are needed to determine the usefulness of KIM-1 as a urinary and serum biomarker for [^177^Lu]Lu-DOTA-(Tyr3)-octreotate induced kidney damage.

A ^177^Lu dose dependent expression of CDKN1A transcript was observed in our previous biomarker studies^18,19,21^. Those findings concur with the CDKN1A expression observed in the present study. A dose dependent expression was also observed for S100A6. ADIPOQ on the other hand, showed only a moderate expression in proximal tubules in kidneys from mice injected with 150 MBq[^177^Lu]Lu-DOTA-(Tyr3)-octreotate with or without A1M. Injection of 90 MBq [^177^Lu]Lu-DOTA-(Tyr3)-octreotate (with or without A1M) did not seem to affect the ADIPOQ expression, indicating that ADIPOQ may not be as sensitive as the other biomarkers investigated in this study.

The weight increase of the animals in the control groups was, according to the animal supplier, within the range of normal weight gain. There was a similar spread within all the groups, and the spread within the treated groups was not greater than in the control groups. Difference in mean weight was observed between the irradiated groups and the control groups, in agreement with previous finding (*28*). Combining 150 MBq[^177^Lu]Lu-DOTA-(Tyr3)-octreotate with A1M did not result in higher mean weight. Combining A1M with 90 MBq[^177^Lu]Lu-DOTA-(Tyr3)-octreotate did result in higher total mean weight, although at the end of the study A1M did not improve the body weight of the mice. Only a few mice were sacrificed prior to the end of the study, and these mice were from different groups (one from the PBS control group) and the reason for early termination was not likely due to radiation induced damage.

At the end of the study, 9 months after injection, no morphological signs of kidney toxicity were observed in mice receiving 90 MBq [^177^Lu]Lu-DOTA-(Tyr3)-octreotate with or without A1M. For mice that received 150 MBq [^177^Lu]Lu-DOTA-(Tyr3)-octreotate with or without A1M, postmortem autopsy indicated abnormal kidneys (enlarged with cyst-like structure). Radiation induced damage was confirmed by morphological signs of kidney injury. Mice with kidney damages had in general higher levels of RBP4 in the urine and higher expression of KIM-1, CDNK1A, NGAL and S100A6 in kidney tissue. This correlation needs to be further studied before it can be established. It is also important to investigate the relationship between urinary RBP4 levels and absorbed dose to the kidneys in humans to verify the potential usefulness in the clinic.

To our knowledge, the only proposed and tested radioprotector for kidneys in radionuclide therapy is amifostine, that was given to rats together with [177Lu]Lu-octreotate^47^. The results were similar radioprotection as with reduction of kidney uptake using lysine, but amifostine has not been applied for clinical use. Instead, more studies have been performed with the intention to reduce the uptake and retention of the radionuclide in kidneys, and several combinations between a drug and lysine have also shown promising results but that still needs to be tested clinically^47^.

## Conclusion

Evaluation of urinary biomarkers for kidney damage confirmed the results from a previous study that RBP4 is a promising early responding biomarker for [^177^Lu]Lu-DOTA-(Tyr3)-octreotate induced kidney damage in mice. The results were less promising for urinary NGAL, creatinine and cystatin C as early responding biomarker. In kidney tissue, KIM-1, CDKN1A and S100A6 showed a dose dependent expression, making them promising markers for [^177^Lu]Lu-DOTA-(Tyr3)-octreotate induced injury in kidney tissue. ADIPOQ on the other hand, do not seem to be as promising. Altogether, no clear long-term protective effect of single dosage of 5 mg/kg A1M on radiation induced effects on kidneys were detected. Further long-term studies are needed to establish the optimum A1M dosage and administration schedule in relation to administration of [^177^Lu]Lu-DOTA-(Tyr3)-octreotate.

## Data Availability

All data are in general presented in the paper, except for all microscopic images. All data is available from the corresponding author on reasonable request.

## References

[1] Hennrich, U. & Kopka, K. Lutathera(^®^): The First FDA- and EMA-Approved Radiopharmaceutical for Peptide Receptor Radionuclide Therapy. *Pharmaceuticals (Basel Switzerland)*. **12**, 10.3390/ph12030114 (2019). 10.3390/ph12030114 PMC6789871 31362406

[2] Melis, M. et al. Localisation and mechanism of renal retention of radiolabelled somatostatin analogues. *Eur. J. Nucl. Med. Mol. Imaging*. **32**, 1136–1143. 10.1007/s00259-005-1793-0 (2005). 15912401 10.1007/s00259-005-1793-0

[3] Sandstrom, M. et al. Individualized dosimetry of kidney and bone marrow in patients undergoing 177Lu-DOTA-octreotate treatment. *J. nuclear medicine: official publication Soc. Nuclear Med.* **54**, 33–41. 10.2967/jnumed.112.107524 (2013). 10.2967/jnumed.112.107524 23223392

[4] Sundlov, A. et al. Individualised (177)Lu-DOTATATE treatment of neuroendocrine tumours based on kidney dosimetry. *Eur. J. Nucl. Med. Mol. Imaging*. **44**, 1480–1489. 10.1007/s00259-017-3678-4 (2017). 28331954 10.1007/s00259-017-3678-4 PMC5506097

[5] Larsson, M. et al. Estimation of absorbed dose to the kidneys in patients after treatment with 177Lu-octreotate: comparison between methods based on planar scintigraphy. *EJNMMI Res.* **2**, 49. 10.1186/2191-219x-2-49 (2012). 23006939 10.1186/2191-219X-2-49 PMC3506567

[6] Geenen, L. et al. Overcoming nephrotoxicity in peptide receptor radionuclide therapy using [(177)Lu]Lu-DOTA-TATE for the treatment of neuroendocrine tumours. *Nucl. Med. Biol.* **102–103**, 1–11. 10.1016/j.nucmedbio.2021.06.006 (2021). 34242948 10.1016/j.nucmedbio.2021.06.006

[7] Ahlstedt, J., Tran, T. A., Strand, S. E., Gram, M. & Åkerstrom, B. Human Anti-Oxidation Protein A1M–A Potential Kidney Protection Agent in Peptide Receptor Radionuclide Therapy. *Int. J. Mol. Sci.* **16**, 30309–30320. 10.3390/ijms161226234 (2015). 26694383 10.3390/ijms161226234 PMC4691176

[8] Åkerstrom, B., Maghzal, G. J., Winterbourn, C. C. & Kettle, A. J. The lipocalin alpha1-microglobulin has radical scavenging activity. *J. Biol. Chem.* **282**, 31493–31503. 10.1074/jbc.M702624200 (2007). 17766242 10.1074/jbc.M702624200

[9] Åkerstrom, B. & Gram, M. A1M, an extravascular tissue cleaning and housekeeping protein. *Free Radic. Biol. Med.* **74**, 274–282. 10.1016/j.freeradbiomed.2014.06.025 (2014). 25035076 10.1016/j.freeradbiomed.2014.06.025

[10] Olsson, M. G. et al. Bystander cell death and stress response is inhibited by the radical scavenger alpha(1)-microglobulin in irradiated cell cultures. *Radiat. Res.* **174**, 590–600. 10.1667/rr2213.1 (2010). 20954860 10.1667/RR2213.1

[11] Ahlstedt, J. et al. Biodistribution and pharmacokinetics of recombinant α(1)-microglobulin and its potential use in radioprotection of kidneys. *Am. J. Nucl. Med. Mol. Imaging*. **5**, 333–347 (2015). 26269772 PMC4529588

[12] Kristiansson, A. et al. Protection of Kidney Function with Human Antioxidation Protein alpha1-Microglobulin in a Mouse (177)Lu-DOTATATE Radiation Therapy Model. *Antioxid. Redox. Signal.*10.1089/ars.2018.7517 (2018). 29943622 10.1089/ars.2018.7517 PMC6477591

[13] Kristiansson, A. et al. (177)Lu-PSMA-617 Therapy in Mice, with or without the Antioxidant α(1)-Microglobulin (A1M), Including Kidney Damage Assessment Using (99m)Tc-MAG3 Imaging. *Biomolecules* **11**, 10.3390/biom11020263 (2021). 10.3390/biom11020263 PMC7916794 33579037

[14] Kristiansson, A. et al. Hematological and renal toxicity in mice after three cycles of high activity [(177)Lu]Lu-PSMA-617 with or without human α(1)-microglobulin. *Sci. Rep.* **14**, 10787. 10.1038/s41598-024-61370-2 (2024). 38734765 10.1038/s41598-024-61370-2 PMC11088679

[15] Ferguson, M. A. & Waikar, S. S. Established and emerging markers of kidney function. *Clin. Chem.* **58**, 680–689. 10.1373/clinchem.2011.167494 (2012). 22311920 10.1373/clinchem.2011.167494 PMC5136473

[16] Bagshaw, S. M. & Bellomo, R. Cystatin C in acute kidney injury. *Curr. Opin. Crit. Care*. **16**, 533–539. 10.1097/MCC.0b013e32833e8412 (2010). 20736828 10.1097/MCC.0b013e32833e8412

[17] Vaidya, V. S., Ferguson, M. A. & Bonventre, J. V. Biomarkers of acute kidney injury. *Annu. Rev. Pharmacol. Toxicol.* **48**, 463–493. 10.1146/annurev.pharmtox.48.113006.094615 (2008). 17937594 10.1146/annurev.pharmtox.48.113006.094615 PMC2742480

[18] Schüler, E. et al. Transcriptional response of kidney tissue after 177Lu-octreotate administration in mice. *Nucl. Med. Biol.* **41**, 238–247. 10.1016/j.nucmedbio.2013.12.001 (2014). 24434014 10.1016/j.nucmedbio.2013.12.001

[19] Schüler, E. et al. Time- and dose rate-related effects of internal (177)Lu exposure on gene expression in mouse kidney tissue. *Nucl. Med. Biol.* **41**, 825–832. 10.1016/j.nucmedbio.2014.07.010 (2014). 25156037 10.1016/j.nucmedbio.2014.07.010

[20] Schüler, E., Parris, T. Z., Helou, K. & Forssell-Aronsson, E. Distinct microRNA expression profiles in mouse renal cortical tissue after 177Lu-octreotate administration. *PLoS One*. **9**, e112645. 10.1371/journal.pone.0112645 (2014). 25386939 10.1371/journal.pone.0112645 PMC4227842

[21] Schüler, E. et al. Potential Biomarkers for Radiation-Induced Renal Toxicity following (177)Lu-Octreotate Administration in Mice. *PLoS ONE*. **10**, e0136204. 10.1371/journal.pone.0136204 (2015). 26287527 10.1371/journal.pone.0136204 PMC4546116

[22] Dalmo, J. et al. Evaluation of retinol binding protein 4 and carbamoylated haemoglobin as potential renal toxicity biomarkers in adult mice treated with (177)Lu-octreotate. *EJNMMI Res.* **4**, 59. 10.1186/s13550-014-0059-x (2014). 26116120 10.1186/s13550-014-0059-x PMC4452688

[23] Levey, A. S. et al. National Kidney Foundation practice guidelines for chronic kidney disease: evaluation, classification, and stratification. *Ann. Intern. Med.* **139**, 137–147. 10.7326/0003-4819-139-2-200307150-00013 (2003). 12859163 10.7326/0003-4819-139-2-200307150-00013

[24] Filler, G. et al. Cystatin C as a marker of GFR–history, indications, and future research. *Clin. Biochem.* **38**, 1–8. 10.1016/j.clinbiochem.2004.09.025 (2005). 15607309 10.1016/j.clinbiochem.2004.09.025

[25] Schrezenmeier, E. V., Barasch, J., Budde, K., Westhoff, T. & Schmidt-Ott, K. M. Biomarkers in acute kidney injury - pathophysiological basis and clinical performance. *Acta Physiol. (Oxford, England)*. **219**, 554–572. 10.1111/apha.12764 (2017). 10.1111/apha.12764 PMC5575831 27474473

[26] Jana, S. M. P. & Roy, S. Proficient Novel Biomarkers Guide Early Detection of Acute Kidney Injury: A Review. *Diseases* **11**, 8 (2022). 36648873 10.3390/diseases11010008 PMC9844481

[27] Griffin, B. R., Faubel, S. & Edelstein, C. L. Biomarkers of Drug-Induced Kidney Toxicity. *Ther. Drug Monit.* **41**, 213–226. 10.1097/ftd.0000000000000589 (2019). 30883514 10.1097/FTD.0000000000000589 PMC6436396

[28] Norden, A. G., Lapsley, M. & Unwin, R. J. Urine retinol-binding protein 4: a functional biomarker of the proximal renal tubule. *Adv. Clin. Chem.* **63**, 85–122. 10.1016/b978-0-12-800094-6.00003-0 (2014). 24783352 10.1016/b978-0-12-800094-6.00003-0

[29] Marchetti, F., Coleman, M. A., Jones, I. M. & Wyrobek, A. J. Candidate protein biodosimeters of human exposure to ionizing radiation. *Int. J. Radiat. Biol.* **82**, 605–639. 10.1080/09553000600930103 (2006). 17050475 10.1080/09553000600930103

[30] Kultova, G., Tichy, A., Rehulkova, H. & Myslivcova-Fucikova, A. The hunt for radiation biomarkers: current situation. *Int. J. Radiat. Biol.* **96**, 370–382. 10.1080/09553002.2020.1704909 (2020). 31829779 10.1080/09553002.2020.1704909

[31] Li, S., Lu, X., Feng, J. B., Tian, M. & Liu, Q. J. Identification and Validation of Candidate Radiation-responsive Genes for Human Biodosimetr. *Biomed. Environ. Sci.: BES*. **30**, 834–840. 10.3967/bes2017.112 (2017). 29216961 10.3967/bes2017.112

[32] Cheng, C. W. et al. Calcium-binding proteins annexin A2 and S100A6 are sensors of tubular injury and recovery in acute renal failure. *Kidney Int.* **68**, 2694–2703. 10.1111/j.1523-1755.2005.00740.x (2005). 16316344 10.1111/j.1523-1755.2005.00740.x

[33] Yamakado, S. et al. Urinary adiponectin as a new diagnostic index for chronic kidney disease due to diabetic nephropathy. *BMJ open. diabetes Res. care*. **7**, e000661. 10.1136/bmjdrc-2019-000661 (2019). 31245009 10.1136/bmjdrc-2019-000661 PMC6557464

[34] Song, S. H. et al. High serum adiponectin as a biomarker of renal dysfunction: Results from the KNOW-CKD study. *Sci. Rep.* **10**, 5598. 10.1038/s41598-020-62465-2 (2020). 32221363 10.1038/s41598-020-62465-2 PMC7101406

[35] Andersson, C. K. et al. Recombinant α(1)-Microglobulin Is a Potential Kidney Protector in (177)Lu-Octreotate Treatment of Neuroendocrine Tumors. Journal of nuclear medicine: official publication. *Soc. Nuclear Med.* **60**, 1600–1604. 10.2967/jnumed.118.225243 (2019). 10.2967/jnumed.118.225243 PMC6836861 30926650

[36] Åkerström, B. et al. rA1M-035, a Physicochemically Improved Human Recombinant α(1)-Microglobulin, Has Therapeutic Effects in Rhabdomyolysis-Induced Acute Kidney Injury. *Antioxid. Redox. Signal.* **30**, 489–504. 10.1089/ars.2017.7181 (2019). 29471681 10.1089/ars.2017.7181 PMC6338582

[37] Schüler, E., Österlund, A. & Forssell-Aronsson, E. The amount of injected 177Lu-octreotate strongly influences biodistribution and dosimetry in C57BL/6 N mice. *Acta Oncol. (Stockholm Sweden)*. **55**, 68–76. 10.3109/0284186x.2015.1027001 (2016). 10.3109/0284186X.2015.1027001 25813472

[38] Rassol, N. et al. Co-administration with A1M does not influence apoptotic response of (177)Lu-octreotate in GOT1 neuroendocrine tumors. *Sci. Rep.* **13**, 6417. 10.1038/s41598-023-32091-9 (2023). 37076494 10.1038/s41598-023-32091-9 PMC10115890

[39] Alattar, A. G. K. A. et al. Recombinant α1-Microglobulin (rA1M) Protects against Hematopoietic and Renal Toxicity, Alone and in Combination with Amino Acids, in a 177Lu-DOTATATE Mouse Radiation Model. *Biomolecules* **13**, 928 (2023). 37371508 10.3390/biom13060928 PMC10296637

[40] Rassol, N. et al. Evaluation of coadministration schedules of rA1M for kidney protection after administration of 177Lu-octreotide. *Radiat. Prot. Dosimetry*. **201**, 995–1005. 10.1093/rpd/ncaf090 (2025). 40875274 10.1093/rpd/ncaf090 PMC12392891

[41] Svensson, J., Molne, J., Forssell-Aronsson, E., Konijnenberg, M. & Bernhardt, P. Nephrotoxicity profiles and threshold dose values for [177Lu]-DOTATATE in nude mice. *Nucl. Med. Biol.* **39**, 756–762. 10.1016/j.nucmedbio.2012.02.003 (2012). 22445743 10.1016/j.nucmedbio.2012.02.003

[42] Forrer, F. et al. From outside to inside? Dose-dependent renal tubular damage after high-dose peptide receptor radionuclide therapy in rats measured with in vivo (99m)Tc-DMSA-SPECT and molecular imaging. *Cancer Biother. Radiopharm.* **22**, 40–49. 10.1089/cbr.2006.353 (2007). 17627412 10.1089/cbr.2006.353

[43] Rolleman, E. J. et al. Long-term toxicity of [(177)Lu-DOTA (0),Tyr (3)]octreotate in rats. *Eur. J. Nucl. Med. Mol. Imaging*. **34**, 219–227. 10.1007/s00259-006-0232-1 (2007). 17021812 10.1007/s00259-006-0232-1

[44] Bober, B. et al. Early Complications of Radioisotope Therapy with Lutetium-177 and Yttrium-90 in Patients with Neuroendocrine Neoplasms-A Preliminary Study. *J. Clin. Med.* **11**, 10.3390/jcm11040919 (2022). 10.3390/jcm11040919 PMC8874379 35207193

[45] Saracyn, M. et al. Renal Disturbances during and after Radioligand Therapy of Neuroendocrine Tumors-Extended Analysis of Potential Acute and Chronic Complications. *Int. J. Mol. Sci.* **24**, 10.3390/ijms24087508 (2023). 10.3390/ijms24087508 PMC10138694 37108668

[46] Summary of Recommendation Statements. *Kidney Int. Supplements* ;**3**:5–14. doi:10.1038/kisup.2012.77. (2013). 10.1038/kisup.2012.77 PMC4284512 25598998

[47] Vegt, E. et al. Renal toxicity of radiolabeled peptides and antibody fragments: mechanisms, impact on radionuclide therapy, and strategies for prevention. *J. nuclear medicine: official publication Soc. Nuclear Med.* **51**, 1049–1058. 10.2967/jnumed.110.075101 (2010). 10.2967/jnumed.110.075101 20554737

